# Neurophysiological insights into impaired mentalization in borderline personality disorder an electroencephalography study

**DOI:** 10.3389/fpsyt.2023.1293347

**Published:** 2024-01-10

**Authors:** Seokho Yun, So-Hye Jo, Hye-Jin Jeon, Bokyung Choo, Jeong-Ho Seok, Hyunkyung Shin, In-Young Kim, Sun-Woo Choi, Bon-Hoon Koo

**Affiliations:** ^1^Department of Psychiatry, Yeungnam University Hospital, Yeungnam University College of Medicine, Daegu, Republic of Korea; ^2^Industry-Academic Cooperations, Yonsei University College of Medicine, Seoul, Republic of Korea; ^3^Institute of Behavioral Science in Medicine, Yonsei University College of Medicine, Seoul, Republic of Korea

**Keywords:** borderline personality disorder, electroencephalography, mentalization, childhood adverse experience, power spectral density

## Abstract

**Introduction:**

Borderline personality disorder (BPD) is characterized by interpersonal and emotional instabilities, recurring suicidal tendencies, and feelings of emptiness. Childhood adverse event is reported in 70%–80% of cases involving BPD. Furthermore, the deficiency in mentalization capacity plays a significant role in emotion dysregulation and social interaction problems within individuals with BPD. This study explored the relationship among childhood adverse experiences, mentalization capacity, and neurophysiological activity in patients with BPD.

**Methods:**

Resting-state electroencephalography was used to identify the neural correlates associated with childhood adversity and mentalization deficits. The participants included 45 patients with BPD and 15 healthy controls.

**Results:**

The BPD group exhibited reduced alpha activity during eyes-closed rest, indicating heightened arousal even during relaxation. Correlations were found between the power spectral density (PSD) and mentalization capacity in the delta and theta ranges, suggesting an association between PSD and emotional awareness and expression. Gamma activity negatively correlated with psychic equivalence, implying a blurring of the boundaries between internal mental experiences and the external world.

**Conclusion:**

These findings offer insights into the pathophysiology of BPD, provide potential diagnostic markers, and suggest personalized treatment approaches based on mentalization traits.

## 1 Introduction

Borderline personality disorder (BPD) is one of the personality disorders characterized by interpersonal and emotional instabilities, persistent feelings of emptiness, impulsivity, and recurrent suicidal behaviors or threats. Some of the symptoms of BPD are similar to those of other psychiatric diseases, such as mood disorders, and are thus difficult to diagnose. In addition, the lack of useful biological markers for diagnosing BPD often leads to delays in its diagnosis. Individuals with BPD experience high levels of psychological distress including dysphoria and tension (1). Moreover, patients with BPD find it difficult to endure such psychological distress (2). Due to emotional crises, individuals with BPD often resort to frequent medical interventions, including hospitalizations, emergency room visits, and medication (3). In addition, patients with BPD have impaired social and occupational functioning, which imposes a socioeconomic burden (4).

Although the exact cause of BPD remains elusive, it is thought that a sensitive and anxious inborn temperament with a biological basis is formed by continuous interaction with socio-environmental risk factors. Multiple complex factors are implicated in the interactions between the individual and the environment, making it difficult to establish the pathophysiology of BPD. However, among the various factors associated with the emergence of BPD, exposure to adverse events during childhood, such as abuse, neglect, and bullying is strongly correlated with the development of BPD (5, 6). A history of childhood maltreatment is observed in approximately 70%–80% of individuals diagnosed with BPD (7). These early experiences can disrupt the development of healthy emotional coping mechanisms and impair the ability to navigate social interactions effectively (8). As a result, individuals who have experienced childhood adversity may exhibit challenges in emotion regulation and social functioning, which are hallmark features of BPD.

Mentalization refers to the cognitive and emotional capacity to understand and interpret one’s own and others’ thoughts, emotions, intentions, and behaviors (9). It involves the ability to consider multiple perspectives, recognize that behaviors are often driven by internal mental states, and make inferences about the mental states of oneself and others (10). In essence, mentalization enables individuals to navigate social interactions, understand motivations, and anticipate others’ actions (11). Difficulties in mentalization are considered a central characteristic of BPD. Individuals with BPD often struggle to accurately understand their own and others’ emotions, intentions, and behaviors (12). This can lead to challenges in forming and maintaining relationships, regulating emotions, and interpreting social cues (10).

Numerous neuroimaging studies, including neurophysiological investigations, have been conducted to elucidate the mechanisms underlying BPD. Brain imaging studies of patients with BPD have reported a characteristic limbic structure, especially volume reduction of the hippocampus and amygdala, compared with structures in healthy control group (13). Another study reported a decrease in frontal lobe volume in patients with BPD, suggesting a decrease in inhibitory function (14). In neurophysiological studies of BPD, attempts have been made to identify the characteristics of BPD using an electroencephalography (EEG) and an event-related potential (ERP). Several studies have reported that frontal alpha asymmetry is more pronounced in the patients with BPD following rejection scenarios (15, 16). Furthermore, Kramer et al. (17) observed that EEG-vigilance regulation presented differently in BPD. However, neurophysiological studies on BPD addressing the childhood adverse events, fundamental causes of BPD onset, and the mentalization challenges have been notably limited (18–20).

Due to the limited research on the relationship between childhood adverse events and mentalization challenges in BPD, this study aimed to explore the relationship between childhood adverse events, mentalization capacity, and neurophysiological activity in individuals with BPD. Specifically, we investigated resting-state EEG measurements to uncover the potential neural correlates associated with adverse childhood experiences and mentalization deficits in patients with BPD.

## 2 Materials and methods

### 2.1 Participants

This study was undertaken as a component of a multicenter study performed at the Yeungnam University Hospital and Gangnam Severance Hospital to assess the effectiveness of structured treatments for BPD. The psychological attributes utilized in the EEG measurements and analysis were acquired during the preliminary screening stage prior to the participants’ involvement in therapeutic programs, such as mentalization-based treatment and structured clinical management (21, 22). We enrolled individuals diagnosed with BPD using the Structured Clinical Interview for DSM Disorders (SCID) (23). The healthy control (HC) group comprised individuals without a psychiatric history or neurological disorders, as determined by SCID. Written informed consent was obtained from all participants. In this study, 45 participants (men = 4) were enrolled in the BPD group and 15 participants (men = 2) were included in the HC group. Fisher’s exact test was used to determine whether there were any differences in sex between the two groups, whereas the Kolmogorov–Smirnov test was used to assess age differences between the two groups.

### 2.2 Assessment tool

To evaluate the characteristics of BPD, we used PROVE, a battery assessment tool developed to screen for protective and vulnerable factors related to mental health. Among the five subcategories of PROVE, we used the adverse childhood experience section (PROVE-ACE) and mentalization capacity section (PROVE-MC). The validity and reliability of the PROVE battery were verified through comparative analysis with standardized scales (24). PROVE-ACE is a scale that quantifies negative experiences such as abuse that individuals experienced in the growing period. It consists of six subcategories: exposure to neglect, bullying, sexual abuse, emotional abuse, physical abuse, and domestic violence during childhood. The higher the frequency and severity of the negative experiences, the higher the score. PROVE-MC evaluates the mentalization ability, that is, understanding and applying knowledge of one’s own and other people’s states of mind (12). Five subcategories were assessed: lack of emotional awareness, emotional expression, mentalizing others, psychic equivalence, and hasty-incomplete mentalizing. An increased score reflected the increasing difficulty of mentalization. Kolmogorov–Smirnov tests were conducted on the six experiences measured by PROVE-ACE to assess the differences in adverse childhood experiences between the two groups. Additionally, to examine the disparities in mentalization capacity between the two groups, Kolmogorov–Smirnov tests were performed on the five capacities measured by PROVE-MC. To address the multiple-comparison problem in both analyses, the Bonferroni correction was applied.

### 2.3 Electroencephalography recording and preprocessing

Resting-state EEG were recorded for 5 min each during eyes-closed (EC) and eyes-open (EO) states in a quiet, isolated environment. EEG recordings were obtained using a 64-channel Geodesic sensor net and an N400 System from EGI (Amsterdam, Netherlands). We preprocessed the data using the EEGLAB module and an in-house MATLAB code (25). We eliminated the first minute of data and conducted our analysis exclusively on a 2 min segment located in the middle of the EEG data. The EEG was resampled at 500 Hz, and high-pass filtering was applied for frequencies above 1 Hz. We used the “clean_rawdata” function in EEGLAB for bad channel rejection and interpolated excluded channels using adjacent channels (26). We referenced the data using the mastoid channels TP9 and TP10 and removed line noise using the “zapline_plus” module in EEGLAB (27, 28). After applying the independent component analysis (ICA) using the “runica” function in EEGLAB, we calculated artifact probabilities of ICA components using the “ICLabel” module (29). Components with a *Z*-score of three or more were discarded. Following ICA component removal, low-pass filtering was applied at frequencies below 30 Hz. Laplacian filtering was employed to enhance the spatial resolution (30). We segmented the EEG every 2 s and removed noise epochs using the “pop_rejepoch” function of EEGLAB. We performed the same preprocessing procedure separately for both the EC and EO states.

### 2.4 Spectral power analysis and statistical assessment

We calculated the power spectral density (PSD) in decibels (dB) of each epoch in the range of all frequencies from 1 to 30 Hz using EEGLAB’s “spectopo” function, which utilizes the Welch’s method (31). A statistical test was performed by averaging the PSD calculated for each state-specific epoch. Independent-sample permutation t-tests were used for all electrodes and frequencies to determine differences between the BPD and HC groups. Statistical significance was verified by a cluster permutation test with FieldTrip in MATLAB (cluster-defining threshold alpha = 0.05, permutation *n* = 500, cluster alpha = 0.05; two-tailed test) (32, 33). The cluster permutation test comprises two primary stages. In the first stage, known as the cluster forming stage, spectral power density values located in voxels within a channel*frequency 2D dimension are transformed into *T*-values. Subsequently, these transformed *T*-values that exceed a predefined cluster defining threshold and are adjacent in either the channel or frequency dimension are aggregated into a single cluster, effectively forming clusters based on these spatial and spectral proximity criteria. In the inference stage, which constitutes the second phase, we evaluate the *t*-values of clusters formed during the forming stage to determine if they exceed a pre-defined cluster threshold. This step is essential to ascertain whether the clusters created are of statistical significance. For the calculation of these two types of *t*-values, nonparametric resampling methods, such as Monte Carlo sampling, are predominantly employed. Furthermore, the Spearman correlation between psychological traits and PSD was computed in the BPD group, and the cluster permutation test was performed with the same parameters. Cluster permutation test effectively addresses the multiple comparisons problem by identifying clusters of statistically significant data points across space and frequency domains, thereby allowing for a more accurate interpretation of neural activity patterns. Therefore, our use of the cluster permutation test goes beyond mere statistical analysis; it is central to our approach in interpreting the results, with a particular focus on the clusters that have been identified (34).

## 3 Results

### 3.1 Demographic data and psychological assessment

The demographic data and psychological assessment results of the BPD and HC groups are summarized in Table 1. No significant differences in age (BPD vs. HC, *p*-value: 26.22 ± 4.69 vs. 26.40 ± 4.08, *p* = 0.58) and sex (men = 4 vs. men = 3, *p* = 0.35) between the two groups were observed. In the PROVE-ACE assessment, four adverse experiences, namely emotional abuse (9.56 ± 3.60 vs. 4.33 ± 3.15, *p* < 0.01), sexual abuse (5.82 ± 8.42 vs. 0.27 ± 0.59, *p* < 0.01), neglect (4.80 ± 6.04 vs. 0.60 ± 0.83, *p* < 0.01), and bullying (10.76 ± 7.51 vs. 3.93 ± 3.35, *p* < 0.01), were significantly higher in the BPD group compared to the HC group. From the PROVE-MC assessment, two aspects of mentalization capacity, namely, lack of emotional awareness (11.31 ± 3.44 vs. 5.73 ± 4.04, *p* < 0.01) and psychic equivalence (5.36 ± 2.27 vs. 1.80 ± 1.62, *p* < 0.01), were significantly higher in the BPD group compared to the HC group.

**Table 1:** Demographic data and psychological assessment of the borderline personality and healthy control groups.

	BPD Group (*n* = 45)	HC Group (*n* = 15)	*p*-value
**Demographic data**			
Gender			0.35
Male	4	3	
Female	41	12	
Age (year)	26.22 ± 4.69	26.40 ± 4.08	0.58
**Adverse childhood experience**			
Emotional abuse	9.56 ± 3.60	4.33 ± 3.15	<0.01*
Physical abuse	12.04 ± 8.37	4.93 ± 3.86	0.25
Sexual abuse	5.82 ± 8.42	0.27 ± 0.59	<0.01*
Neglect	4.80 ± 6.04	0.60 ± 0.83	<0.01*
Domestic violence	10.91 ± 11.82	4.07 ± 4.56	0.13
Bullying	10.76 ± 7.51	3.93 ± 3.35	<0.01*
**Mentalization capacity**			
Lack of emotional awareness	11.31 ± 3.44	5.73 ± 4.04	<0.01*
Lack of emotional expression	8.60 ± 4.23	7.07 ± 2.02	0.26
Psychic equivalence	5.36 ± 2.27	1.80 ± 1.62	<0.01*
Hasty incomplete mentalizing	5.00 ± 3.26	5.00 ± 3.18	0.99
Lack of mentalizing others	4.82 ± 2.58	5.00 ± 1.47	0.83

Data are presented as number only or mean ± standard deviation. Statistical significance was determined using the Fisher’s exact test or Kolmogorov–Smirnov test. The *p*-value represents the uncorrected value, and * indicates variables that showed significant differences after Bonferroni correction.

### 3.2 Group difference of power spectral density

**Figure 1:**
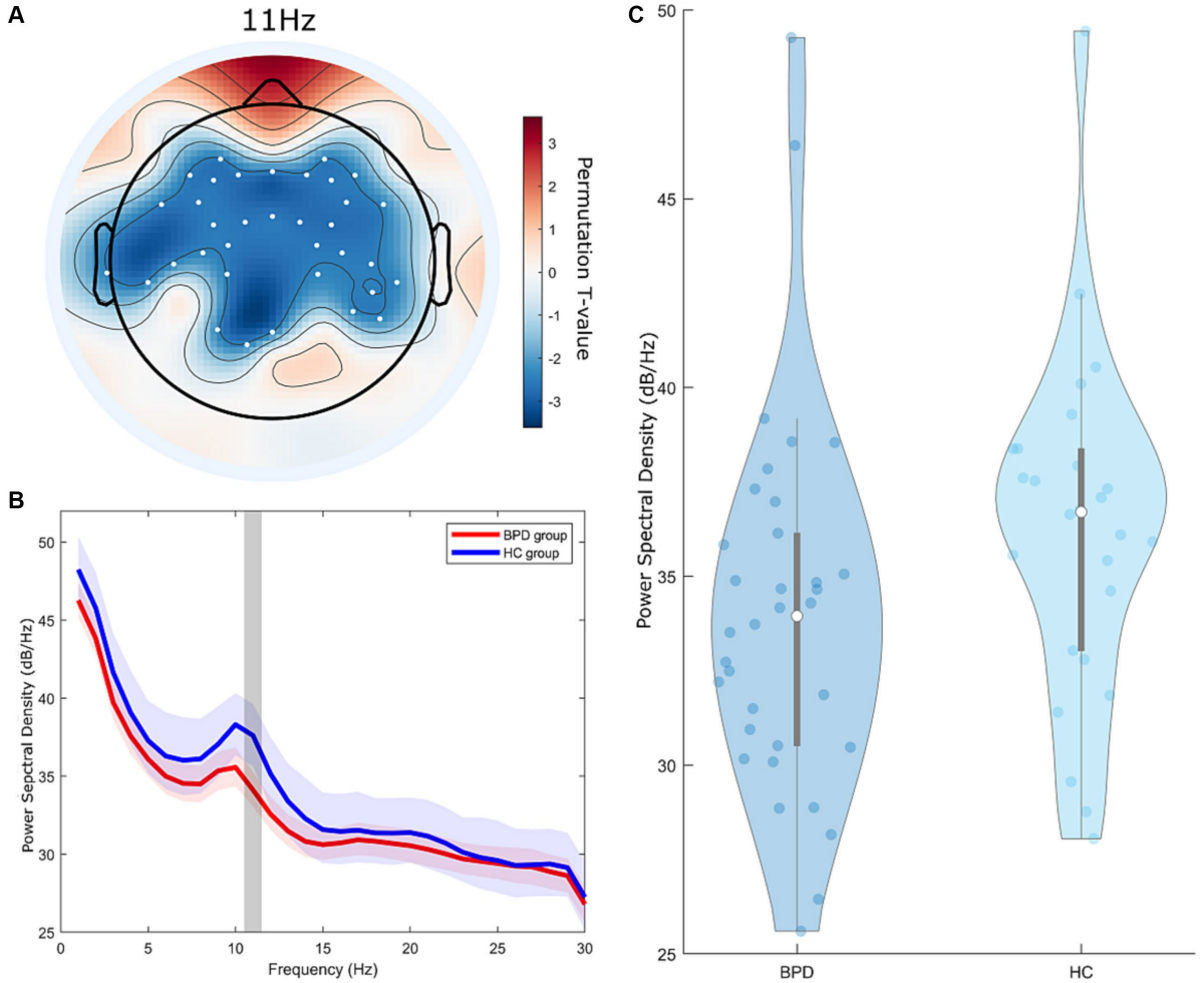
Group differences in power spectral density (PSD) between the borderline personality disorder (BPD) and healthy control (HC) groups at resting eyes-closed state. **(A)** Topographic distribution of significant differences in PSD between the BPD and HC groups, indicated by cluster *t*-values from the cluster permutation test. The colored regions on the topoplot exclusively represent the brain regions at 11 Hz, where significant clusters were formed according to the cluster permutation test. Color intensity indicates the permutation *t*-value. The white dots on the topoplot indicate the positions of the channels that constitute the significant cluster. **(B)** Frequency-dependent average *t*-values of significant differences in PSD between the BPD and HC groups from the cluster permutation test. The lightly shaded area represents the 90% confidence interval of the PSD. The gray zone highlights the 11 Hz area in the frequency domain where a significant cluster is positioned. **(C)** Violin plot of average power spectral density representing significant clusters of PSD differences between the BPD and HC groups from the cluster permutation test.

During the EC state, a significant cluster was identified, indicating a notable difference in PSD between the BPD and HC groups (Figure 1, cluster-*p* = 0.048, *t* = 2.32–2.76, where *t* refers to the *t*-value from the cluster permutation test). Within this cluster, the BPD group had a lower PSD than the HC group. This cluster was observed within the 11 Hz frequency range in the frequency dimension and was found in all brain regions globally, excluding the occipital area, in the spatial dimension.

### 3.3 Associations of psychological characters and power spectral density

**Figure 2:**
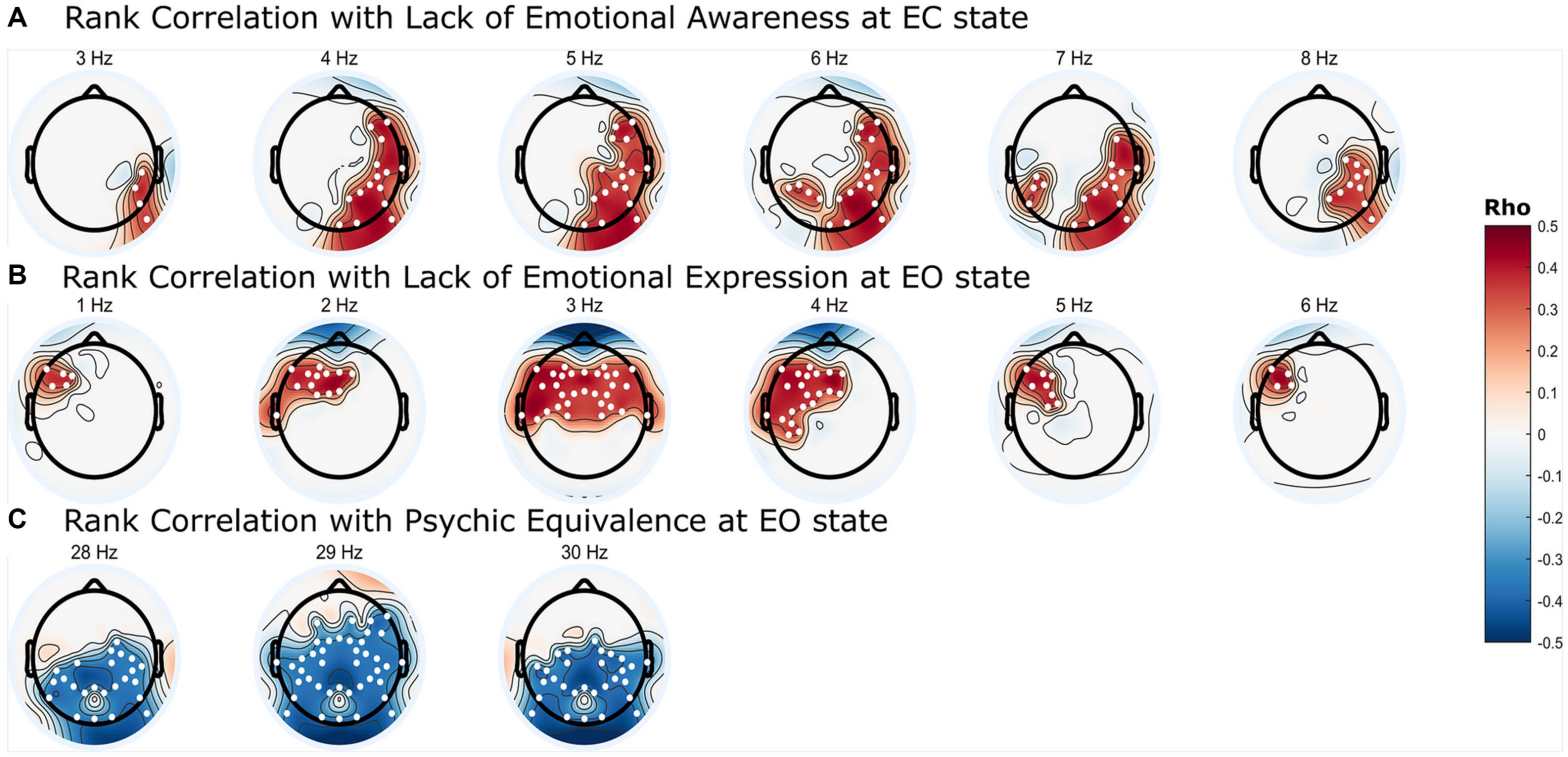
Topographic representation of significant correlation clusters between mentalization capacity and power spectral density (PSD) in borderline personality disorder. **(A)** Frequency-dependent Spearman’s rank correlation coefficients (rho) values between resting eyes-closed state PSD and lack of emotional awareness in mentalization. **(B)** Frequency-dependent rho values between resting eyes-open (EO) state PSD and lack of emotional expression in mentalization. **(C)** Frequency-dependent rho values between resting EO state PSD and psychic equivalence in mentalization. The colored regions on the topoplot exclusively represent the brain regions where significant clusters were formed according to the cluster permutation test, and the intensity of the color intensity correspond to the rho value. The white dots on the topoplot indicate the positions of the channels that constitute the significant cluster.

**Figure 3:**
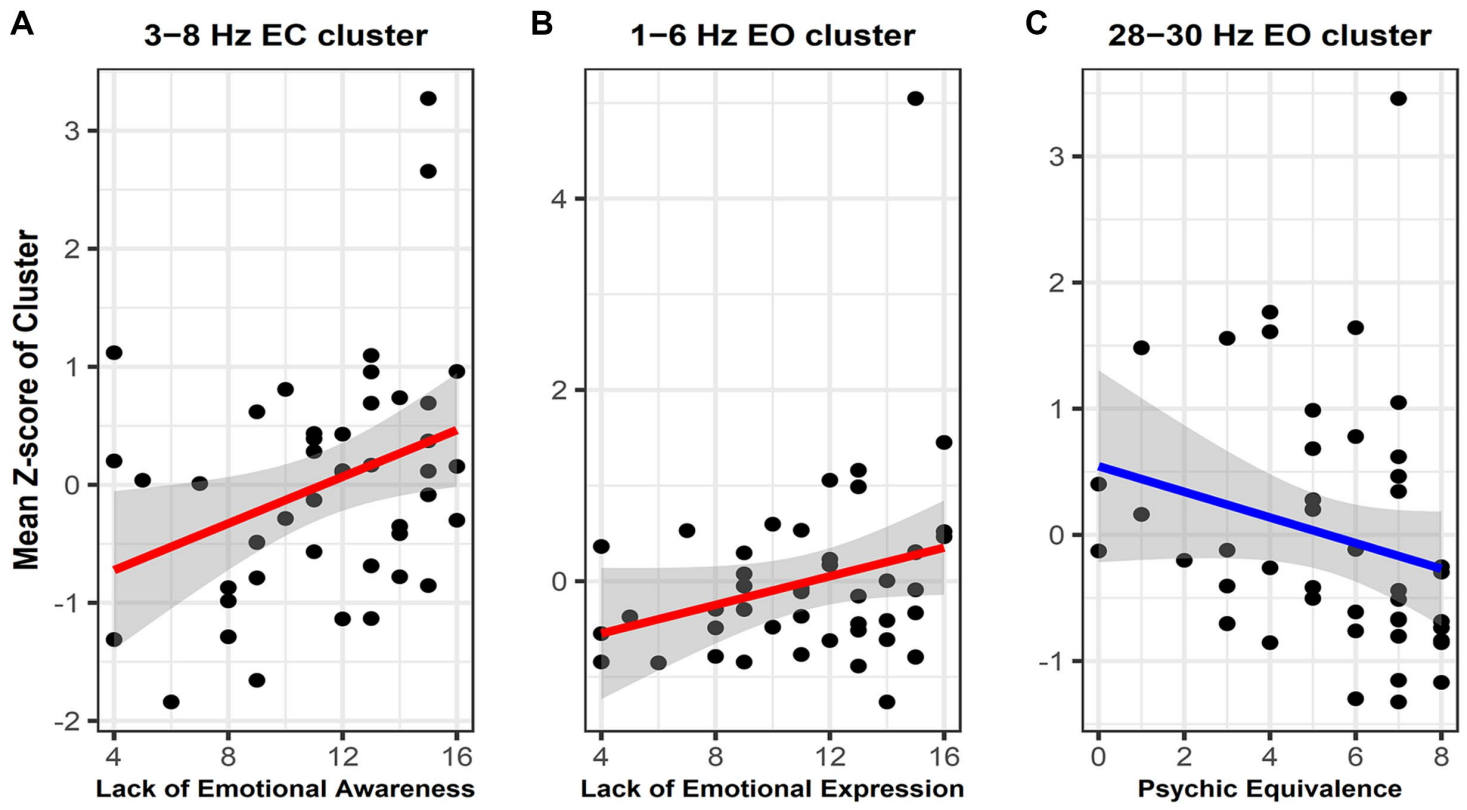
Scatterplot with regression line showing the correlation between the mean power spectral density (PSD) *Z*-scores within significant clusters and mentalization capacity. **(A)** Correlation between lack of emotional awareness and the mean PSD *Z*-score within the 3–8 Hz cluster in the eyes-closed state. **(B)** Correlation between lack of emotional expression and the mean PSD *Z*-score within the 3–8 Hz cluster in the eyes-open (EO) state. **(C)** Correlation between psychic equivalence and the mean PSD *Z*-score within the 28–30 Hz cluster in the EO State. The gray zone represents the area representing the 95% confidence interval of the regression line between mentalization capacity and PSD. Given the varying magnitudes of PSD values across different frequency bands, we normalized these values by converting them into *Z*-scores. We then plotted the mean of these *Z*-scores against the degree of mentalization capacity.

Significant correlation clusters were found between the PSD of the resting-state EEG and three types of mentalization capacity from the PROVE-MC battery: lack of emotional awareness, lack of emotional expression, and psychic equivalence. Lack of emotional awareness showed a significant positive correlation with PSD in the EC state (Figure 2A, cluster-*p* = 0.044 rho = 0.33–0.40, where rho refers to the rank correlation coefficient). This cluster was found in the 3–8 Hz range in the frequency dimension and was located in the right (Rt.) temporal, Rt. parietal, Rt. occipital, and left (Lt.) parietal areas in the spatial dimension (Figure 3A). Lack of emotional expression showed a significant positive correlation with the PSD of the EO state (Figure 2B, cluster-*p* = 0.048 rho = 0.34–0.44). This cluster was situated within the 1–6 Hz frequency range and across both the temporal and parietal areas in the spatial dimension (Figure 3B). Psychic equivalence was significantly negatively correlated with the PSD of the EO state (Figure 2C, cluster-*p* = 0.044 rho = −0.41–0.37). This cluster was found in the 28–30 Hz range in the frequency dimension and in the right temporal area, both parietal areas, and both occipital areas in the spatial dimension (Figure 3C).

## 4 Discussion

### 4.1 Adverse childhood experience and mentalization capacity in borderline personality disorder

In our study, the BPD group exhibited higher scores in four sections—emotional abuse, sexual abuse, neglect, and bullying—than the HC group in the domains evaluating adverse childhood experiences. These findings align with Linehan’s contention that exposure to adverse childhood experiences can hinder the ability to comprehend, manage, or tolerate emotional responses, potentially leading to BPD, which is characterized by difficulty expressing personal emotional experiences (35). Additionally, the BPD group demonstrated significantly higher scores in two out of the five mentalization capacity sections, lack of emotional awareness and psychic equivalence, compared to the HC group. Mentalization encompasses the process of introspecting into one’s own and others’ thoughts, emotions, and minds. This concept highlights that most mental disorders can manifest with some degree of mentalization difficulty (36). Notably, everyone’s ability to understand their own and others’ minds can falter during heightened emotions or stress. In the context of BPD, this vulnerability is particularly pronounced, often leading to intense reactions triggered by minor everyday stress or others’ trivial mistakes (36), which aligns with our findings that the BPD group scored higher on the lack of emotional awareness scale. Furthermore, psychic equivalence scores were also higher in the BPD group. Psychic equivalence is a well-documented characteristic among individuals with BPD, and patients with BPD often exhibit highly specific behavioral patterns, such as being exceptionally aggressive or displaying dependency on particular individuals (37, 38). This phenomenon is hypothesized to contribute to their distinct interpersonal dynamics (39).

### 4.2 Low alpha activity in the borderline personality disorder group

Through cluster permutation tests, we observed that the BPD group exhibited a lower PSD score in the 11 Hz frequency range during the EC state compared to the HC group. This suggests that individuals with BPD display reduced alpha activity in the brain during the EC state. Alpha activity is associated with inhibitory processes that suppress irrelevant information and contribute to inhibitory processing (40). Consequently, alpha activity tends to increase during rest or EC states and decrease during states of arousal. In our study, the observed differences in alpha activity were confined to the EC state, implying that elevated arousal persisted even when patients with BPD closed their eyes. Patients with BPD maintain elevated arousal even during rest and gradually return to baseline levels (41). Post-traumatic stress disorder (PTSD), which shares hyperarousal as a significant pathophysiological element, has also demonstrated decreased alpha activity in various studies using brainwave analysis (42, 43).

Given these results, we cautiously suggest that the observed reduction in alpha activity in the BPD group in our study might suggest heightened levels of arousal, potentially influenced by challenging experiences during developmental stages, and that disorders sharing changes in arousal levels post-trauma often exhibit high comorbidity, overlapping symptoms, and treatment, suggesting a common etiology (44). This prompts us to tentatively link the observed decrease in alpha activity in the BPD group to these related conditions’ common etiology. Furthermore, alpha activity plays a role in inhibiting self-centered processing during social interactions (45). Therefore, the observed reduction in alpha activity among individuals with BPD suggests a potential link to the ongoing challenges in social interactions experienced by individuals with BPD.

### 4.3 Neurophysiology of mentalization capacity

Mentalization capacity significantly influences social interactions and is closely related to the pathophysiology of BPD. Although some literature treats it similarly to the theory of mind, mentalization encompasses not only understanding others’ emotions but also comprehending and expressing one’s own emotions while considering personal emotional states and the external world. Several neuroimaging studies have explored mentalization capacity; however, despite mentalization being an umbrella term encompassing various concepts, research specifically identifying the neural correlates of individual attributes is rare (46). Our study followed Fonagy’s theory and examined neurophysiological aspects of five mentalization capacities in BPD, revealing neural correlates associated with lack of emotional awareness and emotional expression and psychic equivalence.

A lack of emotional awareness reflects the inability to accurately recognize one’s emotions. Our study identified correlations within the delta and theta frequency ranges in the temporal and parietal regions during the EC state, particularly across all theta frequency areas. Resting-state theta activity is strongly associated with emotional awareness (47). The role of theta activity in emotion regulation is well-documented (48, 49). Our findings connect theta activity, particularly in the parietal region, with explicit and implicit emotion processing (50). Lack of emotional expression identified through cluster analysis within the delta and theta frequency ranges in the left frontotemporal and prefrontal regions during the EO state, particularly in the delta frequency areas, suggests a link between emotional expression and delta activity. Delta activity is associated with mental states, such as sleep and unconsciousness, and cognitive processes, such as memory and semantic processing (51). Additionally, delta activity is connected to emotional and motivational drives, which aligns with the motivation-centric nature of emotion regulation and its potential effect on social interaction (52). Considering prior knowledge about the role of delta activity, the observed correlation between delta activity and lack of emotion expression suggests a close association with the motivation-mediated emotion regulation known to be governed by delta activity.

Psychic equivalence is a mental phenomenon wherein the external reality is ignored under the influence of emotional states (53). Our study demonstrated a negative correlation between psychic equivalence and PSD in the 28–30 Hz cluster across both parietal and occipital areas. Initially, our study design aimed to examine activity ranging from the delta band (1–4 Hz) to the beta band (13–30 Hz), leading to a frequency range specification of 1–30 Hz. Interestingly, clusters associated with psychological equivalence emerged specifically at frequencies starting at 28 Hz. The frequency range of the oscillatory bands of neural activity remains a topic of debate, with gamma band frequencies generally considered to be above 30 Hz (54). Nevertheless, some studies suggest the gamma band could start as low as 28 Hz, and given that the clusters did not form in other beta band frequencies, we found it more plausible to attribute the observed clusters to gamma activity, extending beyond beta activity (55, 56). As a result, we decided to concentrate on the relationship between the gamma band and psychic equivalence. Psychic equivalence, a unique mentalization capacity proposed by Fonagy, relates to over-cognition and a sense of certainty, often leading to momentary confusion between one’s internal and others’ mental states in the external world. Although there is currently an absence of dedicated neuroimaging investigations into psychic equivalence, we carefully considered the implications of gamma activity, which facilitates cross-modal integration among diverse cognitive domains (57). This integration spans perception, cognition, behavior, and, notably, emotion awareness (58, 59). Psychic equivalence characterizes an inability to differentiate internal and external contexts. From a cognitive science and computational neuroscience standpoint, psychic equivalence could signify inadequate integration between internal emotional information and external perceptual information, causing an excessive reflection of internal emotional states (60, 61). Thus, we speculate that the observed correlation between gamma activity and psychic equivalence suggests a deficit in appropriate integration, stemming from the insufficiency of gamma activity. However, to solidify our assumptions, further research extending beyond the 28–30 Hz frequency range to the primary gamma activity range of 30 Hz and above is essential.

Unlike mentalization capacity, adverse childhood experiences did not correlate with PSD. This is due to the complexity of factors such as the timing of trauma exposure, parental coping, caregiving environment, and genetic factors, which collectively contribute to the manifestation of various mental disorders, including BPD, depression, PTSD, and bipolar disorder, despite childhood trauma serving as a potential causal factor for these psychopathologies.

### 4.4 Diagnosis and treatment of borderline personality disorder using neurophysiological correlates

The complex and heterogeneous nature of BPD makes its diagnosis challenging, and misconceptions regarding poor treatment outcomes have hindered proper diagnosis and treatment (62–64). Our novel neural correlates offer the potential for deeper insights into the pathophysiology of BPD and the development of objective diagnostic markers using neurophysiological data, mitigating the reluctance of clinicians to diagnose and thus providing more opportunities for appropriate treatment. Moreover, these correlates shed light on the individual elements of mentalization that are problematic in BPD and offer a broader understanding of this concept.

Therefore, our findings have the potential to assist in the development of new diagnostic methods that utilize neurophysiological data for BPD. Additionally, the modulation of alpha-band activity, which is known to influence meditation and neurofeedback, may offer new avenues for BPD treatment (65, 66). By employing traits related to mentalization capacity, structured treatment programs can be designed based on patient-specific mentalization capacity issues. Moreover, these traits provide objective measurements of treatment effectiveness (36, 67).

### 4.5 Limitations and further studies

This study had certain limitations that warrant consideration. First, the control group had a relatively small sample size, particularly of men, which may have affected the generalizability of the findings. Additionally, focusing exclusively on treatment-seeking individuals may not fully represent the broader population of those with BPD. These limitations necessitate caution when interpreting and extending our findings. Future research should strive for a more diverse participant pool and improved methodologies to ensure comprehensive outcomes across different diagnostic and treatment scenarios. Furthermore, the study did not account for participants’ medication use and coexisting medical conditions, potentially limiting the interpretation of results. Future studies should incorporate these variables to provide a more comprehensive understanding. Despite the significance of emotional neglect in BPD, the PROVE battery employed in our study does not provide a detailed categorization of neglect types. For future studies, it is considered necessary to utilize a assessment that offers a more comprehensive assessment of varied forms of neglect. In our methodology, the use of 64-channel ICA on 2 min of data presents certain limitations due to its short duration. To overcome these limitations in future research, we plan to analyze data of sufficient length, ensuring a more comprehensive and reliable assessment. Moreover, this study analyzed only resting-state EEG data. Future research could directly investigate mentalization processes using ERP during emotional processing and social interactions. Such investigations may elucidate the psychological complexity of BPD and contribute to the development of effective treatment approaches. Additionally, in further research, it is anticipated that an analysis should be conducted regarding the comorbidity of PTSD or complex PTSD and the history of childhood adverse events, in relation to our findings on alpha activity. Lastly, in future research, we plan to investigate whether there are changes in the mentalization capacity of BPD patients, as well as in their neurophysiological activations, following therapeutic programs like MBT.

## Data availability statement

The raw data supporting the conclusions of this article will be made available by the authors, without undue reservation.

## Ethics statement

The studies involving humans were approved by Institutional Review Board, University of Yeungnam University (2021-02-046-011) and Yonsei University (3-2021-0095). The studies were conducted in accordance with the local legislation and institutional requirements. The participants provided their written informed consent to participate in this study.

## Author contributions

SY: Conceptualization, Data curation, Formal analysis, Investigation, Methodology, Visualization, Writing – original draft, Writing – review & editing. S-HJ: Conceptualization, Data curation, Writing – review & editing. H-JJ: Conceptualization, Data curation, Writing – original draft. BC: Conceptualization, Data curation, Writing – review & editing. J-HS: Conceptualization, Data curation, Writing – review & editing. HS: Conceptualization, Data curation, Writing – review & editing. I-YK: Data curation, Writing – review & editing. S-WC: Conceptualization, Data curation, Writing – review & editing. B-HK: Conceptualization, Funding acquisition, Methodology, Writing – review & editing.
